# Violence Against Women: Experiences of Eritrean Refugee Women in Britain

**DOI:** 10.1177/10778012231220372

**Published:** 2023-12-15

**Authors:** Samson Maekele Tsegay, Shewit Tecleberhan

**Affiliations:** 1 School of Education, 152391 Anglia Ruskin University, Cambridge, UK

**Keywords:** refugee, violence against women, gender-based violence, domestic violence, Eritrea

## Abstract

There is a dearth of research on violence against women and girls among refugees, particularly in their host countries. Therefore, informed by a feminist theoretical framework and semistructured interviews, this study explores violence against women focusing on Eritrean refugee women's experiences in Britain. The findings suggest that Eritrean refugee women experience various types of violence, which have short- and long-term effects on their lives. Moreover, the data indicate that host and origin countries’ socioeconomic and cultural situations shape the experiences of refugee women. The research aims to better understand violence against women among refugees and thus improve refugee women's experiences.

## Introduction

Women and girls face various types of violence due to their gender identity or biological sex (Bellizzi & Molek, 2021). It is important to note that all men, women, and other gender minorities experience violence (Colucci & Montesinos, 2013; World Health Organisation [WHO], 2021). However, it mainly affects women due to patriarchal cultures, which affect their social and economic positions in societies (Ajygin, 2010; UNHCR, 2022; Walker, 1999). For instance, about 30% of women in the world experience physical and/or sexual violence in their lifetime (WHO, 2021). Furthermore, even though violence against women could be more prevalent in developing countries, such as sub-Sahara Africa and among refugees and asylum seekers, it affects women in all parts of the world regardless of their geographical location, educational qualification, or income level (Bellizzi & Molek, 2021; Indira & Vijayalakshmi, 2015; Tsegay, 2022a). This study explores violence against refugee women with a particular focus on its causes and effects among Eritrean refugees in Britain.

Refugees cross international borders to escape conflict and persecution. Their asylum applications are often accepted upon confirming that they face a threat of persecution and lack of protection in their own countries (UNHCR, 2011). By 2018, there were about 25.9 million and 3.5 million refugees and asylum seekers, respectively (UNHCR, 2018, 2019). Women and girls comprise around 50% of refugees, internally displaced, or stateless populations (IOM, 2014; UNHCR, 2020). There is also a significant number of refugee women in Europe. For instance, women and girls constituted about 32% of the total asylum application population in the European Union from 2009 to 2018 (Eurostat, 2019).

The United Kingdom hosts many refugees and asylum seekers from various countries, most of whom live in Britain. The UNHCR (2022) reported that there are “135,912 refugees, 83,489 pending asylum cases, and 3,968 stateless persons” in the United Kingdom as of 2021. However, it is also important to note that the United Kingdom has made a deal to send some asylum seekers, who entered the country through the British channel, to Rwanda (Sen et al., 2022). Our study focuses on Eritrean refugee women's experiences for various reasons. Eritrea, a country with an estimated population of 5 million people, has been one of the world's top 10 countries of origin for asylum seekers in the past decade (UNHCR, 2016, 2018). In addition, in the last decade, Eritreans have been among the top ten asylum-seeking nationalities in the United Kingdom (Home Office, 2015; Sturge, 2019; Walsh, 2019). Some of these refugees are women who traveled a long and risky journey to reach their destination countries, expecting social, economic, and political emancipation (Tsegay, 2022b).

Moreover, Eritrean women come to the United Kingdom through family reunions, marriage, or similar means (Tsegay, 2022b). Yet many refugee women are subjected to mistreatment, exploitation, and harassment (Kawar, 2004; Palumbo & Sciurba, 2018), some of the abuses coming from fellow migrants, particularly their intimate partners (Tsegay, 2022a). Furthermore, there is disturbing news about Eritrean refugee men killing their intimate partners (e.g., see Hopperstad, 2020; Wijnen, 2018). Nevertheless, there is a dearth of research on violence against women among refugees, particularly in their host countries. Various studies also suggest that there is a need for research on migration studies that put family, including violence against women, at the center (Choi, 2019; Jang et al., 2014).

Therefore, informed by a feminist theoretical framework and semistructured interviews with 15 Eritrean refugee women in Britain, this study explores refugee women's experiences of violence against women. The overarching research questions that guide the study are (a) what are Eritrean refugee women's experiences of violence against women in Britain; (b) what are the causes of violence against women among Eritrean refugees; and (c) how does violence against women impact refugee women? The study aims to provide a comprehensive understanding of violence against women among refugees and thus improve refugee women's experiences in their host countries.

## Understanding Violence Against Women and Girls: Causes and Consequences

Violence against women and girls (VAWG) is any act of gender-based violence that causes suffering or harm to women and girls (Bellizzi & Molek, 2021; Carastathis, 2014; Crenshaw, 1991; Davis, 2008). Women and girls experience various forms of violence, including physical, sexual, psychological, and economic violence. Physical violence is when a person controls another one through physical force, such as kicking or hitting (Hadi, 2017). Psychological violence comprises verbal abuse, defamation, coercion, gaslighting, and harassment that cause mental and emotional harm to a victim (Aguirre et al., 2020). Economic violence occurs when a perpetrator engages in activities that cause financial damage to a victim (Latcheva, 2017). Economic violence includes denying or limiting access to financial resources, damaging property, and refusing to pay for education, health, and alimony. The WHO report indicates that one out of three women globally have encountered physical and/or sexual violence from their partners or other individuals (WHO, 2021). Ott (2021) further stated that such violence is inflicted through rape, sexual harassment, physical beatings, and homicide (Robbers et al., 2016).

Moreover, research indicates that femicide is another deadly manifestation of VAWG (Obinna, 2021; Sela-Shayovitz, 2010). Defined as “the deliberate killing of a woman by a man because of her gender” (Obinna, 2021, p. 808), femicide is one of the leading causes of death for women in many countries such as South Africa, El Salvador, Guatemala, and Honduras (Abrahams et al., 2013; Obinna, 2021). Sela-Shayovitz (2010) further stated that women are most likely to be killed by their intimate partners or close family members. However, the distribution of intimate partner femicide is not equally distributed in society as it is highly influenced by cultural and structural factors (Landau & Rolef, 1998; Sela-Shayovitz, 2010). For instance, the study by Landau and Rolef (1998) shows that intimate femicide rates in Israel are higher among immigrants, which suggests that traditional patriarchal as well as marginalized communities are more vulnerable to femicide (see also Hadi, 2017; Langmead, 2016; Stewart, 2005).

### Causes

Various factors, such as patriarchy and poverty, contribute to VAWG (Indira & Vijayalakshmi, 2015; Phillimore et al., 2022). The key and common cause of VAWG is patriarchy, a system of institutionalized societal values that place women below or subordinate to men (Hadi, 2017). Patriarchy, particularly in developing countries, is exercised through gender segregation, restrictive codes of behavior, and ideologies to force women to conform to traditional family and cultural values (Tsegay, 2022a). Hooks (2010, pp. 44–45) stated:

Much feminist theory that critically examines constructions of masculinity shows that to make boys into patriarchal men, society trains them to value silence over speech. They may find themselves becoming people who either cannot talk or, when they talk, can only engage in a monologue. These are the people who talk at us, who by refusing to converse, promote and maintain a hierarchy of domination wherein withholding gives one power over another person.

Patriarchy in all societies allows men to abuse and inflict violence on their partners and children as a form of discipline, punishment, and dominance (Indira & Vijayalakshmi, 2015). Many men also apply cultural norms that offer higher privilege to men in the diaspora, particularly in a situation when women have lower levels of education, access to employment, and social support (WHO, 2021). This aligns with the statement that gender relations in migrant families are shaped by the home and sending countries’ institutions (Nawyn, 2010). Moreover, women with partners experiencing anxiety, depression, and other mental health problems are more likely to face violence (Sánchez et al., 2020). Furthermore, Connell and Messerschmidt (2005) used the concept of “hegemonic masculinity” to explain gender relations, particularly the dominance of men and the subordination of women. They argued that hegemonic masculinity enacts a pattern of practices (e.g., it constructs dominant, aggressive, and violent models of masculinity) that allow the continuation of men's dominance over women (see also Donaldson, 1993). These situations are not different among refugees and migrants, mainly those coming from traditional patriarchal societies like Eritrea. However, it is important to note that not all men engage in harmful hegemonic masculinity, although many show a “complicit masculinity” as they “receive the benefits of patriarchy without enacting a strong version of masculine dominance” (Connell & Messerschmidt, 2005, p. 832).

Unstable socioeconomic and political environments also exacerbate VAWG (Phillimore et al., 2022). For instance, women are at a higher risk of being physically and sexually assaulted during war and conflict (Kostovicova et al., 2020; Stark & Ager, 2011). In addition, research indicates that women and girls coming to Europe through illegal routes, such as the Mediterranean route, face a high risk of VAWG on the journey to their destination countries (Bellizzi & Molek, 2021). Furthermore, poverty is another prominent cause of VAWG. Refugee women and girls, as one of the most marginalized, powerless, and impoverished people in the world, including in economically developed Western countries (Langmead, 2016; Stewart, 2005), face VAWG even when they reach their destination (Keygnaert et al., 2015; Phillimore et al., 2022). Many refugee women lack necessities and are forced to engage in prostitution for money or food to feed their families (Bellizzi & Molek, 2021; Hadi, 2017; Phillimore et al., 2022).

Moreover, young girls, who are pushed into early or forced marriages without finishing their education, are more likely to become victims of VAWG at the hands of their partners (Phillimore et al., 2022). Although some families arrange such marriages thinking it benefits girls, particularly during war and uncertain times, it often results in VAWG. This is because the girls lack the economic capital to negotiate their relationship with their partners and leave the marriage if they want to. Hence, they fail to speak up and break the pattern of abuse as they feel they have nowhere to run. In some cases, they do not report the assaults due to tremendous social pressure, mainly from families, friends, and religious clergies (Indira & Vijayalakshmi, 2015). Some also fear losing the financial assistance they receive from their partners (Whiting, 2016).

### Consequences

Migration makes women, young girls, and boys more vulnerable to violence. It puts them in a situation where they can be exploited by individuals, including those who are supposed to help or protect them (Keygnaert et al., 2015). Hence, such harm or violence affects the victims’ physical, mental, or/and emotional conditions. For example, many refugees and asylum seekers work in poor conditions with little pay (Langmead, 2016; Stewart, 2005). These conditions exacerbate the refugees’ poor economic situation (Mashiri, 2013) and result in other forms of violence, such as mental and emotional violence (Keygnaert et al., 2015).

VAWG causes physical injuries affecting the physical and emotional well-being of the victims (Heise et al., 2002). Women are often bruised and sometimes exposed to severe injuries that claim their lives (Tsegay, 2022a). In addition, sexual violence causes sexually transmitted diseases and unwanted pregnancy (Bellizzi & Molek, 2021). Physical and sexual violence mainly cause injuries which often need medical attention. However, in most cases, women suffer in silence since they do not want to report their perpetrators for social or economic reasons (Indira & Vijayalakshmi, 2015).

Furthermore, VAWG causes severe mental problems such as depression and anxiety (Heise et al., 2002). Most of the victims feel embarrassed to talk about their experiences or seek help after an assault. They become depressed and frustrated to the extent of isolating themselves from society and, in some cases, they commit suicide (Bates et al., 2021). VAWG also causes women and girls to have strained relationships with family members, intimate partners, and friends. It affects the victim's self-confidence and trust in men, including, not surprisingly, their male partners.

In conclusion, gender-based violence affects everyone regardless of gender, class and socioeconomic status. However, it is more prevalent among women, girls, and other vulnerable groups, including refugees. Moreover, patriarchy being the leading cause, various other factors, such as poverty and conflict, exacerbate VAWG, resulting in physical, psychological, and emotional injuries.

### Feminist Theoretical Perspective

Various studies have examined the relevance and contribution of feminist theories to understanding international migration and violence against women and girls in particular (Altamirano, 1997; Nawyn, 2010). Moreover, the inclusion of gender in analyzing migration research has made feminist theories significant in understanding migrants’ experiences (Nawyn, 2010). Therefore, this study applies feminist theory to understand gender inequality among refugees and examine violence against refugee women.

Feminism is a movement which consists of socioeconomic and political movements and ideologies to end women's exploitation and subjugation by destabilizing systems of power and oppression (Chaudhury et al., 2017; Hooks, 2000). It focuses on “gender” as the essential organization of society in socioeconomic and political aspects and understanding reality. Furthermore, many feminist scholars, such as Kimberlé Crenshaw, argue that women's oppression is caused by an intersection of various factors, including gender, race, class, culture, religion, and geographical location (Carastathis, 2014; Crenshaw, 1991; Davis, 2008). This approach suggests examining multiple, converging or interwoven systems of oppression to understand women's experiences (Tsegay, 2022a).

Feminist movements have campaigned for women's equality and freedom from oppression. Some focused on women's ability to attain equal political and legal rights by uplifting themselves in academic and socioeconomic arenas; whereas others demanded a “drastic reordering of society in which male supremacy is eliminated in all social and economic contexts” (Chaudhury et al., 2017, p. 112). However, although their efforts have been successful in some sectors and countries, many women, particularly refugees, still face gender-based violence, particularly from their intimate partners (Tsegay, 2022a). Therefore, informed by feminist theory, this study aims to better understand and thus tackle violence against women among refugees.

## Methodology

### Data Collection

The study employed a qualitative research approach based on semistructured interviews conducted with Eritrean refugee women living in Britain. First, an open call was circulated to (networks of) Eritrean refugees using telephone calls, social media, and other communication channels. We also used various platforms (such as YouTube and Clubhouse) to inform them about the research project and call for participants. Then, purposive sampling was used to select participants with a range of features to address the case under study (Silverman, 2013). Accordingly, 15 Eritrean refugee women were selected considering their VAWG experiences, marital status, educational level, employment status, and years of residence in Britain (see Table 1).

**Table 1.** Participants’ Profiles (at the Time of the Interview).

No	Name of participant (pseudonym)	Sex	Age	Marital status	Educational qualification	Employment	Children
1.	Aymi	F	20–25	Single	High school	Unemployed	-
2.	Sinit	F	30–35	Married	Undergraduate degree	Full-time	3–4
3.	Stela	F	30–35	Married	High school	Full-time mom	3–4
4.	Yosan	F	30–35	Married	College student	Student	-
5.	Liya	F	30–35	Married	High school	Full-time mom	1–2
6.	Amber	F	36–40	Divorced	High school	Full-time	1–2
7.	Haben	F	36–40	Married	Certificate	Full-time mom	1–2
8.	Senait	F	36–40	Married	High school	Full-time	3–4
9.	Linda	F	36–40	Married	Diploma	Part-time	3–4
10.	Semhar	F	36–40	Separated	High school	Full-time	1–2
11.	Hanna	F	36–40	Separated	Undergraduate degree	Full time	1–2
12.	Genet	F	41–45	Separated	High school	Full time	1–2
13.	Almaz	F	41–45	Separated	Primary school	Full-time mom	5–6
14.	Fozia	F	41–45	Married	High school	Full-time mom	1–2
15.	Elen	F	51–55	Separated	High school	Full-time mom	3–4

Although the primary target was to interview Eritrean refugee women who had lived in Britain at least for one year in order to ensure that they have a basic understanding of their host country (e.g., culture and rights), most of the study participants had been in the country for 5 or more years. The interviews were conducted from June to August 2022 via Zoom, Microsoft Teams, and telephone calls to provide greater flexibility for the research team and the participants. The interviews were audio-recorded with the permission of the interviewees and transcribed into English to better code and anlayze the data. The recruitment and interviews did not account for the participants’ English level, but we, the researchers, are fluent in Tigrigna and English. The participants were also asked if they wanted to be interviewed by a woman. Therefore, except for one, all the interviews were conducted in Tigrigna due to the participants’ choice or English capacity; and most of the interviews were carried out by a woman. The interviews took about 35 minutes each.

### Data Analysis

Thematic data analysis was applied to analyze the data collected through semi-structured interviews as it summarizes the key features and offers a rich interpretation of the data (Braun & Clarke, 2006, 2016; Bryman, 2008). Thematic analysis also allows us to systematically categorize and analyze the data from the stories of the migrants in order to seek commonalities, relationships, and any other patterns that address the research questions (Silverman, 2013). It identifies and describes patterns of experience and the overarching design that unites them (Ayres, 2008). In particular, we followed Braun and Clarke's (2006) six phases of conducting thematic analysis: familiarize with the data, generate initial codes, search for themes, review themes, define and name themes, and write up. Accordingly, after transcribing the interviews, we read the transcripts several times to familiarize ourselves with the data and generate codes. Then, we reorganized the codes into themes and analyzed the data with a reference to the research questions and literature.

### Ethical Considerations

The research project adheres to the ethical guidelines of Anglia Ruskin University (ARU) and the British Educational Research Association at all stages. Most of the participants were concerned about the confidentiality of their interviews. Hence, specific attention was given to confidentiality, anonymity, and informed consent throughout the project. We obtained consent from the participants after providing them with information and allowing them to ask any questions about the project. In addition, we kept the digital recordings and transcripts in a secure, password-protected area, accessible only to the research team. The transcripts were also anonymized, and details enabling the participants’ identification were removed. The participants’ names were replaced with pseudonyms to maintain confidentiality and anonymity. Ethical approval was obtained from ARU.

## Experiences of Eritrean Refugee Women

### Trends of Violence Against Eritrean Refugee Women

Many Eritrean refugee women in Britain experience financial, physical, sexual, and psychological violence, particularly from their intimate partners. Although no reason can be legitimate to harm women or anyone else, this study revealed that multiple factors contribute to violence against women.

#### Financial Violence: “Men Want to Control Everything”

Refugees are one of the most marginalized and impoverished people in the world (Langmead, 2016; Stewart, 2005). The current study also suggests that refugees face difficulty managing the meager resources they get from their income, including government benefits. In most cases, family relations are patriarchal. Thus, men control the financial resources and act as head of the family. With women taking care of children and house chores, they are forced to quit their jobs or work part-time, whereas men work full-time and earn more money than their partners (Alakeson, 2012). As Sinit puts it, such a situation creates a financial imbalance between men and women in the family. The study participants argued that economic inequality is often created when men think that the money they earn belongs solely to them instead of the entire family, whereas trying to control any resources gained through their partners’ income or the Universal Credit. Sinit and Amber, who lived in Britain for more than a decade, further noted:

In most cases, men work and control the household's financial issues. Some women do not know how much money they (the family) have, which sometimes leads to misunderstanding and conflict. It also brings financial or economic violence. (Sinit)

Men want to control everything, including their wives; they want them to be at home. They solely control the money they earn; they control the children's benefits and any other money given to the family and allocate a small amount of money for their wives to buy groceries and other necessities. Sometimes, the men buy these supplies themselves to control the money and their wives’ activities. (Amber)

The data indicates that many Eritrean refugee women have financial constraints to support themselves and their families because they usually take care of the household responsibilities and have no financial arrangements with their partners. The problem with such partners is that they try to put all the money, including the child benefits, into their accounts and use it as they see fit without consulting their partners. For example, Almaz, who moved to Britain for a family reunion, stated that her partner was using the money for gambling. Sinit also shared that she has come across fellow refugees asking about “the amount of child benefits and the duration it is given” because they do not control or have a say in the families’ financial matters.

Haben stated that the root cause of financial violence is traditional Eritrean culture which “gives men higher authority in a family.” Additionally, women's economic dependence on their partners is at the center of their control and subordination (Tsegay, 2022a). Men could not continue their authority in the diaspora without disrupting women's financial security by disallowing them to work and controlling their finances.

Universal Credit decreases the dependency of women on their husbands. It also reduces women's tolerance toward their (abusive) husbands. They feel that their Universal Credit would support them and start asking themselves, ‘why would I need a man if I am not happy, if he is not collaborating with me, or if he is not helping me as much as he can’. (Sinit)

Some men want to control the children's benefits and their partners. However, women who do not work because of parental responsibilities want the money to be off-limit. They want the money to be spent on their children, as they should be, without their partners’ control. The children's benefit usually becomes a source of conflict and violence, such as verbal violence. (Elen)

As indicated above, many of our participants noted the significance of financial management or independence. They argued that there should be a common understanding of how a family's finances should be managed. For example, Hanna and Fozia noted that it is unfair to beg their husbands for money to buy essential household supplies. In addition, the study findings revealed that many Eritrean refugee women have to move to part-time jobs or interrupt their careers to take care of their children (Tsegay, 2022a). For instance, Stela interrupted her job to look after their three children but gets minimal financial support from her partner who has a full-time job.

Moreover, Sinit, Liya, and Haben echoed Elen's point that financial violence often develops into psychological and physical violence. In addition, they suggested that many men consider household responsibility as light work and often fail to recognize or appreciate their partners’ chores. Nevertheless, as Sinit and Almaz noted, these women rarely socialize with other people as they spend most of their time at home with their children or are not motivated by their partners to make friends. Hence, they become lonely and vulnerable to mental health problems.

#### Physical Violence: “It Is *Rooted in Cultur*e”

Physical violence is another situation that many Eritrean refugee women experience in Britain. The findings indicate that many refugee women are often physically threatened and beaten by their partners. Such toxic masculinity is mainly associated with traditional Eritrean culture, which tolerates wives beating by their partners (see also Indira & Vijayalakshmi, 2015; Tsegay, 2022a). For example, Almaz was a victim of physical violence for more than 2 years. Moreover, Haben and Sinit described:

The main reason is our traditional upbringing which gives men higher authority in a family. Women usually are supposed to agree with what men decide. However, men find it difficult to continue their dominance in the diaspora because many women work and have financial security. Therefore, women do not usually agree with what men (their partners) decide and want. This develops into conflict, starting with minor insults and then to beating. (Haben)

The way we grew up has made women tolerate wife beating. Sometimes, women are beaten by their husbands in front of their children. The way we grow up influences our life. In our culture, women are the oppressed ones. Even we have proverbs that encourage constant abuse of women (by men), including ‘*bi’eray ab melsi, sebeyti ab salsti*’, one that suggests a woman should be beaten twice a week. (Sinit)

The above excerpts indicate the impact of culture on violence against women. In particular, the proverb shows the perception of men on violence against their intimate partners. Most of our participants and their partners were born and raised in Eritrea. Eritrean culture has a considerable impact on their lives. Although they have lived for a long time in Britain, men found it difficult to give up their dominance over their partners and create a family based on shared decisions (Tsegay, 2022a). Instead, they demand to be respected by their partners while failing to do the same. Sometimes, they use physical violence to enforce dominance over their partners. Culture is not only significant but also deep-rooted in people's lives. Hence, it takes time to unlearn certain harmful practices and develop new identities (Kim, 2001). In addition, Sinit, Semhar, Almaz, and Senait stated that religious institutions advise women to tolerate their husbands, including their abusive behaviors. Senait, who had lived in Britain for about 10 years, emphasized that “religious institutions are part of the (patriarchal) system that contribute to women's oppression.”

Various sources indicate that women are being killed by their intimate partners (see Hopperstad, 2020; Wijnen, 2018). The majority of our participants reflected on these pieces of news while discussing physical violence. They opined that even though wife beating is common in Eritrea, killing your partner is out of the question. For instance, Sinit stated, “Eritrean society is based on patriarchal structure. Still, in our tradition, men beat their wives, but this does not go up to killing.” Nonetheless, participants such as Almaz argued that there is a close connection between partner killing and patriarchal culture, which allows men's dominance and tolerates violence over their partners. To justify this argument, Almaz asked, “why do we not hear about women killing their partners, but it is almost common for men to kill their partners”? Research shows that women worldwide are more likely to be the victims of homicide (Abrahams et al., 2013; Obinna, 2021; Sela-Shayovitz, 2010), with about 58% killed by intimate partners or family members (United Nations Office on Drugs and Crime, 2018). The number of intimate femicides is also concerning in the United Kingdom. For example, 52% of the 110 women killed by men in 2020 were killed by their current or former partners (Allen et al., 2020).

#### Sexual Violence: “Unspoken Crime”

Sexual violence is a crime regardless of who commits it. Research shows that sexual violence is the most prevalent form of VAWG, affecting 69.3% of female migrants, including refugees, entering Europe (Robbers et al., 2016). This is very high compared to 11% of sexual violence experienced by European girls or women (Robbers et al., 2016). However, sexual violence is not openly spoken about, especially if the perpetrator is an intimate partner. Only two participants (Amber and Genet) in this study mentioned sexual violence in the interviews. Amber and Genet came to Britain a decade and a half ago through a family reunion and the Mediterranean Sea route, respectively. After divorcing her partner, Amber tried to look for a romance and start a family. Nevertheless, she said that the one she thought would be her future partner wanted to rape her.

We spoke for some time, and then I invited him to my house for coffee. However, he tried to rape me. I struggled to protect myself but could not match his physical strength. Then, I told him he could do whatever he was planning to, but I will go directly to the police. Then he stopped; probably, he was afraid of the consequences. (Amber)

Genet also described that her friend's husband tried to touch her private parts and kiss her by force.

He forced himself on me. He wanted to kiss me on my lips just outside of a lift. I tried to stop him, but he was not willing to stop until he saw someone coming. I have not told this to anyone, even to his wife. (Genet)

Research shows that women in general and migrant women in particular experience more sexual harassment and victimization than men (Hadi, 2017; Kawar, 2004; Palumbo & Sciurba, 2018). The above excerpts also indicate many untold stories of women regarding sexual violence. Amber and Genet did not report to the police because reporting could portray them as the cause of someone's imprisonment, and they could be humiliated for being victims of sexual assault. This is mainly associated with the notion that they should not give away their fellow countrymen to the police. In addition, it is related to the misconception among some people that sexual violence is the victims’ fault for instigating men's sexual desires through provocative dressing styles or behaviors (Feiring & Taska, 2005; Hadi, 2017; McElvaney et al., 2021). Genet described this as, “either way, we [women] lose” because she also thought that not reporting to the police could motivate the perpetrators to assault other women.

It is important to note that sexual violence is not the victims’ fault (Hadi, 2017). Hence, it is not the victim's fault if perpetrators are sent to prison, but the consequences of their actions. Overall, the data indicates that most women participants were uncomfortable reporting their perpetrators, particularly if they were their intimate partners, close friends, or family members.

#### Psychological Violence: “Some Words Hurt More Than Physical Abuse*”*

Eritrean refugee women face psychological violence inflicted through verbal abuse, defamation, coercion, gaslighting, and harassment that causes mental and emotional harm. In addition, the participants stated that the power imbalance between men and women encourage men to manipulate and mistreat their partners. In most cases, such violence harms the women's emotional well-being and makes them doubt themselves and any decision they want to make to emancipate themselves from patriarchal oppression.

Many men use offensive language against their partners. They do not respect their partners when they are talking. Sometimes, they insult them, which relates to how we are raised and behave toward a woman. It has less to do with men's psychological or mental issues. (Amber)

Men expect their partners to recognize their work as demanding or exhausting, but they do not give back such recognition to women's work at home. In such circumstances, some women get angry and dissatisfied. Women also need their work to be recognized; they need people to understand that women work hard at home with their children and all the household chores. (Sinit)

Amber's statement suggests that verbal abuse shows the perpetrator's lack of respect for the victims. She also argued that the psychological violence inflicted upon Eritrean refugee women by their intimate partners is more connected to how the men are raised rather than the posttraumatic stress disorder they are exposed to due to their dangerous journey from Eritrea to Britain. Amber, Genet, and Aymi stated that traditional Eritrean culture cultivates hegemonic masculinity, which legitimizes men's dominance over women (Connell & Messerschmidt, 2005). In addition, Yosan and Aymi noted that the stress caused by the migrants’ journey and the negative experiences they encountered in their destination countries have a significant impact on their personality, including their irritability or offensive behaviors (see also Tsegay, 2022b; Zembylas, 2012). Research also suggests that women with partners experiencing stress, anxiety, depression, and mental health issues are more vulnerable to violence (Sánchez et al., 2020). Furthermore, as Sinit and Senait stated, Eritreans are not good at asking for counseling support. Many feel that counseling is for those who have gone mad. Hence, they do not receive help at the right time for mental health issues, which could lead to uncontrollable behavior and violence against women.

Sinit's excerpt indicates that women, with the main occupation of caring for their family, work very hard at home, but their work is seen as minimal or gets less recognition when compared to working men. Most participants opined that it is particularly tough in Britain because they lack parental support. In addition, children in Britain need more attention than those in Eritrea, who usually play outdoors with their mates without parental supervision. However, in Britain, children need extra care and support and must be accompanied to a playground and other activities.

Moreover, many participants noted that they need competent partners who work hard and care about their families. However, they stated that most men partners are unhappy to be challenged or sit for discussion when they display irresponsible behaviors. In most cases, they respond to women's critique with verbal or physical violence. Elen, who separated from her husband due to physical and psychological violence, noted that “some words (insults) hurt more than physical abuse” as they could leave a long-term negative impact on the victim. Some participants further stated that their partners did not allow them to socialize as socialization could increase awareness and end their mistreatment and oppression. Above all, isolating partners from their friends and family is a form of coercive control and hence psychological abuse (Aguirre et al., 2020). For instance, Almaz, who separated from her husband after a quarter-century of marriage, explained:

My husband used to gamble with his friends. At times, he used to come home angry from outside and displace his rage on me. He was not thinking about our children or their needs and did not want me to talk about it. Also, he did not allow me to socialize—join my friends or go to church—because he feared that I would speak about our condition and get help to end the violence, including the beating I was enduring. (Almaz)

Sinit also stated, “In our culture, the man has a bigger role in the family. He is always respected to the level that a woman (his partner) cannot challenge his arguments or decisions.” Almaz's and Sinit's points suggest that for a woman to argue with her partner is culturally unacceptable or considered rude. Moreover, Almaz's statement indicates the influence of gambling and other related habits such as alcohol and substance misuse on violence against women. Many Eritrean refugees, particularly men, still depend on a tradition that silences women in navigating family or gender relations. Furthermore, the study findings indicated that Eritrean refugees rarely communicate with each other to peacefully solve their problems (see also Tsegay, 2022a). The lack of communication is associated with patriarchy, which values “silence over speech,” because they are not ready to lose hierarchy and men's domination over women (Hooks, 2010, p. 44).

Finally, some participants pointed out that some Eritrean refugee women commit psychological violence against their partners. However, although the participants reiterated that refugee women are more vulnerable to abuse and mistreatment, researchers should also consider men's experiences.

### Factors Contributing to Violence Against Eritrean Refugee Women

Violence against women is affected by socioeconomic, cultural, educational, and personal factors. As indicated above, patriarchal values contribute to women's violence, mainly at the hands of their intimate partners. In addition, the findings suggest that other factors, such as educational qualification and personal behavior, influence the prospect of violence against women. Sinit witnessed:

In most cases, women's awareness about the services available and their usage is related to their educational qualifications and employment. For example, those who have completed their undergraduate degree in Eritrea or here in the United Kingdom have a good English capacity which could help them access information and understand the support available. Hence, the possibility of violence against such women is lower than against those with little or no education. (Sinit)

For instance, Almaz and Elen did not get the chance to complete secondary school. Hence, they lacked linguistic and other capital to access the essential services and integrate into their host country, which often creates a conducive environment for the perpetrators (Tsegay, 2022a).

My problem started in Eritrea and continued here when I came to Britain for a family reunion. Lack of education and awareness is one of the causes of violence against women. I used to tolerate him (my partner and his abusive behavior) for various reasons, but it started to affect my children. He began to beat not only me but also our children. After tolerating him for 2 years and 5 months (in Britain), I began to say enough is enough. I decided to be separated because I thought it would be better for my children and their future. I am happy with that decision. (Almaz)

Violence also emanates from early marriage as both partners do not know each other well. I knew my partner for a very short period before our marriage. Later, I tried to tolerate all his behaviors for the sake of our children, but it did not work because he became violent physically and verbally, and his behaviors threatened my life and my children. It is better to be alone than with a violent partner. **(**Elen)

Educational qualification plays a significant role in helping women protect themselves from abuse and mistreatment. Research indicates that higher academic qualifications allow refugees to get jobs and access social services (Bradatan & Kulcsár, 2014; Fokkema & De Haas, 2011). It is also less compatible with traditional gender roles, which place women at home as housewives (Tsegay, 2022a). Moreover, the above quotes show that many violent relationships end in separation or divorce.

Furthermore, the data revealed that many Eritrean refugee women stay in abusive relationships for a long time for various reasons: children, societal expectations, and lack of awareness (see also Pugh et al., 2021; Whiting, 2016). For example, as seen above, Almaz explained that “lack of education and awareness” was part of why she could not decide to leave her abusive partner for about two and a half years. Almaz also reiterated that she paid a heavy price for her six children to have a “father figure” although her partner was not supporting the children and the family at large. Senait, Semhar and Stela further noted that societal expectations of respecting the man deemed the “head of the family” significantly contribute to their tolerance of abusive relationships. Additionally, Senait and Semhar described:

Many women lack awareness about violence, except for physical abuse. They consider financial abuse, controling, and emotional damage as normal. Many even do not have bank accounts and do not have any say about the money the families get from the Universal Credit. (Senait)

Many women tolerate violence coming from their intimate partners. They do not report to the police or tell anyone, which limits their opportunity of getting support. Some even justify the violence with different reasons—my partner is doing this because he loves me, he cares about me, or it is for my sake. (Semhar)

Many Eritrean refugee women do not have adequate awareness of violence against women. This is because, in Eritrea, women and society generally consider a certain kind of violence against women, such as wife beating, acceptable or normal (Indira & Vijayalakshmi, 2015; Tsegay, 2022a). In this line, Almaz's and Senait's points suggest that refugees and refugee women, in particular, need information about their rights and responsibilities when they reach their destination. Some men exploit their partners’ low level of knowledge about British laws and mistreat them.

Moreover, although migrating to Britain gave women socioeconomic opportunities, violence against women is still prevalent among Eritrean refugees. Many participants opined that refugees lack parental support, which affects violence against women. Sinit and Linda reiterated the role of parents in advising their children and solving conflicts peacefully.

(In Eritrea), when women feel uncomfortable or threatened by their partners, they go to their parent's house until things get solved. However, here (in Britain) there are no such circumstances. There is no parent's house to go to and calm down until things improve. Hence, the partners keep arguing, which sometimes turns to violence. (Sinit)

Migration has a lot to do with exacerbating violence. For example, refugee women need time to understand their host country's culture, laws, and traditions. On top of that, women find it painful to be mistreated by the one they thought would support them because they have no brother or sister to rely on. Things also become worse if the women cannot speak English. (Linda)

Many other participants also echoed the above points, which indicate the impact of migration on violence against women. For example, Amber stated that there were times when the police used her partner as an interpreter for her complaint against him. However, there are no such language barriers in Eritrea. Yet, the perception of Eritrean society that women should submit to their husbands or tolerate certain mistreatment hinders women from leaving abusive relationships before causing severe physical and emotional damage.

### Effects of Violence Against Eritrean Refugee Women

Violence severely impacts the victims’ physical, social, and emotional well-being (Heise et al., 2002; Keygnaert et al., 2015). For instance, the data indicate that economic and psychological violence causes mental and emotional damage since the women feel distressed and vulnerable.

Violence often starts with a small thing like insulting, and if we do not tackle the smallest part of violence, it grows and might put the woman's life at risk. There is some support for gender-based violence victims from the (British) government and Eritrean community activists. However, the time women leave the abusive relationship and get help is significant for their health and well-being. (Haben)

Many women tolerate their abusive partners and stay in the relationship for a long time before separation or divorce. However, as seen above, Haben's quotation implies that such tolerance could endanger women's mental and emotional well-being if they did not deal with the problem in time. This suggests that women should deal with abusive relationships before affecting their health and well-being.

Moreover, physical violence can cause injuries and other damage, which makes the home an unsafe space for women (Sánchez et al., 2020). In addition, such experience creates fear in the women to the extent that they cannot report it to the police.

We (abused women) want to inform the police and deal with abusive husbands, but we are afraid of the consequences. We are so scared of what they could do to us after telling the police. I returned from the police station three or four times without informing them because I was afraid that he would kill me. After all, the killing of women partners has become common among Eritreans. (Almaz)

Despite the fear of leaving her partner, Almaz also understood that staying for long in an abusive relationship would worsen her situation and future relations.

If you do not decide to leave before it damages you, it affects your life and your children. You cannot be sure about having another man in your life, particularly if you have children. It means your life is dedicated to your children as a single person (single mom). There is also fear, which hinders you from traveling freely. (Almaz)

Many participants echoed Almaz's idea regarding the long-term effects of violence. They used to fear for their lives even after separation or divorce, which limited their movements. Elen and Haben also stated that abusive relationship damages women's confidence and self-esteem. For example, Elen and her children were physically and emotionally abused by her partner (and the father of her children). In addition, she noted that verbal abuse could cause long-term damage to the victim.

There is support from police, refugee organizations, and others which help victims of gender-based violence in terms of counseling and other services. I know a woman abused by her partner who got support from these institutions, but it was not enough. The psychological and emotional damage affected the woman's confidence and self-esteem. She could not even properly support her children which were finally taken by the social services. (Haben)

As indicated above, violence has a long-term impact on women's social and emotional well-being. In such cases, they require proper mental and emotional support, including trauma support services. However, as Sinit described, “We (Eritreans) do not often ask for information or seek support.” Similarly, Amber commented, “we do not access all the services available in this country.” These statements indicate that the short and long-term impact of violence against women could be more severe on refugee women than those born or raised in this country. Furthermore, failing to get essential help could cause mental health problems, leading to suicidal behaviors (Kavak et al., 2018), which are more prevalent among ethnic minorities, refugees, and immigrant women (Colucci & Montesinos, 2013).

Gender-based violence affects women in different ways. It affects not only the women but also their children. They do not feel confident to start another life after divorce or separation. Gender-based violence also affects women mentally and might push women to think about committing suicide. (Haben)

Violence against women also has a significant impact on children because any conflict within a family affects the children socially, academically, and psychologically. Moreover, beating or disrespecting women in front of children sends a wrong message to the children. Sinit pointed out, “If your father was respecting your mother, there is a tendency that you would respect your wife. The opposite is also true.” Violent partners can hurt their children and teach them lousy behavior, which might affect the children's future relationships.

Although some women try to tolerate abusive relationships for various reasons such as social, economic, and child reasons, they eventually get separated or divorced. As indicated above, most of the study participants stayed in abusive relationships for some time, hoping their partners would change their behaviors. However, they finally said, “Enough is enough” (Almaz) or “It is better to be alone than with a violent partner” (Elen). Moreover, the socioeconomic and emotional support that the women get significantly influences their road to emancipation from violent relationships. The women's English capacity and knowledge of available services are paramount. Women with children also need financial help to take care of their families and hence depend on government benefits and/or start working. Finally, the participants commented that refugees, more than anyone else, should support each other because they are far from their country of origin, where most or all of their family members live.

## Conclusions

Many Eritrean refugee women in Britain experience violence from men and various factors contribute to this situation. Refugee women face financial, physical, and psychological violence, mostly from their intimate partners. Moreover, the findings suggested that sexual violence is prevalent among Eritrean refugees. However, women do not often report sexual violence or talk about it due to factors associated with a feeling of shame and/or protecting the perpetrators. The study further indicated that most of the contributing factors to violence against women are shaped by the host and origin countries’ socioeconomic and cultural situations (Nawyn, 2010). The cultural or traditional perceptions that view men as the head of the family encourage men to enforce control of their family through either persuasion or coercion. Many women also stay in abusive relationships for lack of awareness, social pressure, or for the sake of their children. In addition, lack of parental support and educational qualifications contribute to the prospects of violence against women. Some women fear that leaving their partners could lead to economic limitations or further violence. Nevertheless, the data indicates that women leave toxic relationships sooner or later. In such cases, the socioeconomic and legal support Britain provides, particularly to victims of gender-based violence, contributes to women's emancipation from patriarchal oppression.

The current study also revealed that violence against women has a significant social, physical, emotional, and psychological impact on women. Eritrean women are often advised and pushed to stay (or try harder) in their relationships, including abusive ones, by their families and religious leaders. In most cases, society perceives women who leave their partners as hysterical. However, this study suggests that the duration for which women stay in abusive relationships has a notable impact on their lives. Women who have been victims of gender-based violence for a long time could face life-threatening injuries or mental health problems leading to suicidal behaviors (Kavak et al., 2018). The data further indicated that domestic violence affects children socially, academically, and psychologically.

The study suggests that violence against women is a concerning matter among Eritrean refugees in Britain. Women have got socioeconomic empowerment in the country. However, Eritrean families mostly follow traditional practices that put men at the top of the family. As Sinit said, “we (Eritreans) are physically here in Britain, but still living in an Eritrean situation.” On the other hand, it is important to recognize women's increased awareness of gender-based violence and opposition to patriarchal domination. Many women used to consider certain kinds of violence, such as wife beating, from their partners as normal. Nevertheless, many stated that they gradually changed their mind. The findings imply a clash of cultures between traditional Eritrean culture and contemporary British values that focus on individual liberty and equality.

This research helps to better understand violence against women among Eritrean refugees. Thus, it contributes to tackling gender-based violence and improving refugee women's experiences. However, as it is done with limited funding and timing, the number of participants and the scope of the study are limited. Hence, further research is required to strengthen the finding, tackle gender-based violence, and improve refugee women's lives. Similar research is also needed to explore violence against refugee men to provide a comprehensive understanding of the issue.
